# Relationships between Physical Activity, Work Ability, Absenteeism and Presenteeism in Australian and New Zealand Adults during COVID-19

**DOI:** 10.3390/ijerph182312563

**Published:** 2021-11-29

**Authors:** Jayden R. Hunter, Rebecca M. Meiring, Ashley Cripps, Haresh T. Suppiah, Don Vicendese, Michael I. Kingsley, Brett A. Gordon

**Affiliations:** 1 Holsworth Research Initiative, La Trobe Rural Health School, La Trobe University, Bendigo 3552, Australia; Michael.kingsley@auckland.ac.nz (M.I.K.); b.gordon@latrobe.edu.au (B.A.G.); 2 Department of Exercise Sciences, Faculty of Science, University of Auckland, Auckland 1023, New Zealand; rebecca.meiring@auckland.ac.nz; 3 Movement Physiology Research Laboratory, School of Physiology, University of the Witwatersrand, Johannesburg 2193, South Africa; 4 School of Exercise and Health Sciences, University of Notre Dame Australia, Fremantle 6160, Australia; ashley.cripps@nd.edu.au; 5 Sport and Exercise Science, School of Allied Health, Human Services and Sport, La Trobe University, Melbourne 3086, Australia; H.Suppiah@latrobe.edu.au; 6 Department of Mathematics and Statistics, La Trobe University, Bundoora 3086, Australia; don.vicendese@unimelb.edu.au; 7 Melbourne School of Population and Global Health, University of Melbourne, Carlton 3053, Australia

**Keywords:** exercise, physical activity, coronavirus, productivity, employee, work ability, health promotion

## Abstract

Public health movement and social restrictions imposed by the Australian and New Zealand governments in response to the COVID-19 pandemic influenced the working environment and may have affected health behaviours, work ability, and job performance. The aim of this study was to determine the associations between health behaviours and work ability and performance during COVID-19 restrictions and if health behaviours were related to demographic or population factors. A cross-sectional survey was used to gather responses from 433 adult employees in Australia and New Zealand between June and August 2020. The survey requested demographic information and used the International Physical Activity Questionnaire, Work Ability Index, and the World Health Organisation’s Health and Work Performance Questionnaire. Multivariate regression models were used to explore relationships between the identified variables while controlling for several possible confounders. Being sufficiently physically active was associated with higher reported physical (aOR = 2.1; *p* = 0.001) and mental work abilities (aOR = 1.8; *p* = 0.007) and self-reported job performance (i.e., lower presenteeism) (median +7.42%; *p* = 0.03). Part-time employees were 56% less likely (*p* = 0.002) to report a good or very good mental work ability. Those with existing medical conditions were 14% less likely (*p* = 0.008) to be sufficiently active and 80% less likely (*p* = 0.002) to report rather good or very good physical work ability. Being sufficiently active was associated with higher physical and mental work abilities and better job performance during the COVID-19 pandemic. Employers should support opportunities for regular physical activity and provide specific support to individuals with medical conditions or in part-time employment.

## 1. Introduction

In response to coronavirus disease (COVID-19), a contagious condition first identified in December 2019, some world governments put in place stay-at-home orders and other movement and social restrictions to minimise the spread of the virus. Movement restrictions varied between New Zealand and Australian states and territories as both countries pursued a suppression or elimination policy, with restrictions including the temporary closure of public spaces and gyms, shops, businesses, schools, and tertiary institutions. In addition, many employees were encouraged or required to work from home, with only essential workers able to continue transiting to the workplace. This is likely to have resulted in changes to employment and health behaviours. Findings from studies across a range of countries indicate that community-wide PA participation decreased [1,2], sedentary behaviour increased [1,3], and poorer mental wellbeing was associated with lower activity levels during this time [1,2,4,5]. These behaviours have important health ramifications, as regularly engaging in sufficient amounts of physical activity (PA) and minimising sedentary behaviour is beneficial for health and important for preventing and managing chronic diseases [6,7]. The World Health Organization recommends that adults engage in 150–300 min of moderate-intensity or 75–150 min of vigorous-intensity PA (or a combination of both) each week, with muscle-strengthening exercise performed on at least 2 days [8]. Even before the pandemic, however, less than one half of all adults globally met these guidelines [9], and in Australia, only 15% of adults reported meeting both the aerobic and strength exercise guidelines on a weekly basis [10,11]. This poses a serious public health problem, as physical inactivity is a leading cause of chronic disease, morbidity, and premature mortality and adds to the global economic burden [12].

Based on previous research, it could be theorised that the reduction in PA during the COVID-19 pandemic could negatively impact work ability and job performance. In an occupational context, physical inactivity is estimated to cost INT$14 billion in lost productivity globally each year [12] and is associated with lower physical and mental work ability [13,14]. However, systematic reviews conducted before COVID-19 reported that regular exercise may increase work ability [15] and reduce absenteeism and presenteeism [16]. These are important findings, as productivity losses due to chronic disease-related absenteeism (i.e., poor health resulting in sick leave) and presenteeism (i.e., when a person at work is unable to perform at full capacity due to illness, stress, or other issues) represent 10–15% of global economic output [17]. Recent evidence suggests a favourable relationship between higher MVPA and lower absenteeism when accounting for various health factors. Observational (each weekly hour of reported PA was associated with the equivalent of 1.2 days per year lower sickness absence amongst Spanish university employees [18]) and interventional data [19] demonstrate positive outcomes for both absenteeism and presenteeism following weekly vigorous intensity aerobic and strength exercise. While findings are not ubiquitous [15], there is evidence demonstrating improved physical and mental work ability following exercise interventions [20,21]. Exercise, therefore, has the potential to mitigate the chronic disease risk associated with sedentary occupations, reduce employee absenteeism and presenteeism, and improve work ability [16]. However, whether relationships between PA, work ability, and job performance exist when various community-wide movement and social restrictions are in place is unknown.

The aim of this study was to investigate if Australian and New Zealand employees who were meeting aerobic and strength exercise guidelines reported higher work ability and lower work-related absenteeism and presenteeism than physically inactive employees during the COVID-19 pandemic, which was being managed with various movement and social restrictions. 

## 2. Materials and Methods

### 2.1. Participants and Recruitment

A cross-sectional study design was used to collect self-reported data. Australian and New Zealand adults were invited to participate in an online survey using REDCap (version 10.0.29, Vanderbuilt University, Nashville, TN, USA) a secure, web-based software platform designed to support data capture for research studies [22,23]. The survey was advertised through La Trobe University, University of Auckland, and University of Notre Dame Australia research newsletters and social media networks, local news media, and online community noticeboards from 9 June–9 August 2020. This recruitment period was selected to maximise the response of a broad representation of the targeted population, and multiple avenues of participant recruitment were deliberately chosen to engage a diverse range of participants from broad demographic backgrounds. A total of 456 respondents completed the survey. During this period, different movement and social restrictions were in place across Australia’s states and territories, placing various limitations on group sizes for gathering indoors and outdoors.

In New South Wales in June, people could meet outside in groups of up to 20, up to 100 people were allowed inside gyms, and indoor gym classes allowed up to 10 people, providing there was only one person per four square meters. From 1 July, restrictions eased such that there was no upper limit on the number of people allowed at an indoor venue, with the four square meter rule still in place. In Victoria, outdoor exercise classes were limited to 10 people, with no sharing of equipment and 1.5 m distancing between people. From 30 June, residents across Melbourne and surrounding postcodes entered a period of lockdown and were only allowed two hours of outdoor exercise per day with one other person or with household members, while indoor exercise venues were closed. On 2 August, a State of Disaster was declared, with metropolitan Melbourne moving into Stage 4 restrictions, which included no travel further than 5 km from home without a work permit. In Western Australia, non-work indoor gatherings allowed up to 100 people at any one time per single undivided space, up to a total of 300 people per venue, providing there was only one person per two square meters. New Zealand was at Alert Level 1—Prepare, with no restriction on personal movement.

Australian and New Zealand residents aged 18 years or older who were employed at the time of the survey were eligible to participate and provided their informed consent before commencing. No other inclusion or exclusion criteria were applied. The survey collected information on participant demographics and characteristics, including gender, age, ethnicity, marital status, education and employment history, height, weight, medical history, and dependent relationships. The survey also collected information on current health-related lifestyle behaviours, including smoking, alcohol consumption, PA, sleep quantity, and information on work ability, absenteeism, and presenteeism. The study was approved by the La Trobe University Human Research Ethics Committee (HEC10200) and is reported according to the STROBE Statement [24] (Appendix A). Data were collected and managed using the REDCap electronic data capture tool hosted at La Trobe University.

### 2.2. Physical Activity and Exercise Participation

Participants responded to 11 questions about their PA and sedentary behaviour over the past week. This information was collected using the short-form International Physical Activity Questionnaire (IPAQ) [25] and strength exercise questions that have been used in previous epidemiological research [10]. Moderate-vigorous intensity aerobic physical activity (MVPA) participation was calculated as weekly energy expenditure (MET-min·wk^−1^) using the validated IPAQ formula [26], and strength exercise participation was reported as the number of days in the past week that the participant performed muscle strengthening exercises. Aerobic and strength exercise participation were compared to current MVPA (i.e., ≥500 MET·min·week^−1^) [8,27] and strength exercise (≥2 days each week) [8] guidelines, respectively. Sedentary behaviour was reported as time spent sitting on a typical weekday during the last seven days.

### 2.3. Other Lifestyle Behaviours

Participants were asked whether or not they were a current smoker or if they had quit in the last six months and how often (if ever) they consumed alcohol. Participants were also asked how many hours of actual sleep they got at night on average over the last month, after reporting their usual bedtime, wake time, and the duration taken to typically fall asleep.

### 2.4. Work Ability

Three questions from the Work Ability Index [28] were included. The first question, validated as a single item [29], asked participants to rate their current work ability compared to their lifetime best using an 11-point scale ranging from 0 (cannot work) to 10 (lifetime best). The next two questions asked participants to rate their current physical work ability with respect to the physical demands of their work and their current mental work ability with respect to the mental demands of their work, each using a 5-point scale ranging from 0 (very poor) to 4 (very good). To enable participants to respond accurately, Tengland’s (2011, p. 275) definition of work ability was provided: “Having the occupational competence, the health required for the competence, and the occupational virtues that are required for managing the work tasks, assuming that the tasks are reasonable and that the work environment is acceptable” [30].

### 2.5. Absenteeism and Presenteeism

Absenteeism and presenteeism (constructs of employee productivity) over the past month were measured using the validated World Health Organisation’s Heath and Work Performance Questionnaire following the provided instructions [31]. To measure absenteeism, participants reported their expected weekly hours of work and their actual number of hours of work over the past month after accounting for time off due to health reasons. This was converted to a measure of relative absenteeism, expressed as the difference between actual hours worked and expected hours of work, as a fraction of expected hours of work, and expressed as a percentage. Negative values indicated that participants worked more hours over the past month than what was expected of them. To measure presenteeism, participants were asked to rate their overall job performance on the days they worked over the past month using an 11-point scale ranging from 0 (total lack of job performance) to 10 (no lack of job performance), which was then expressed as a percentage (0–100%). Presenteeism (i.e., lack of job performance) was calculated as the difference between the reported score and 100%, with higher presenteeism values indicating poorer job performance.

### 2.6. Statistical Analyses

Univariate (unadjusted) and multivariate (adjusted for potential confounding variables including age, gender, ethnicity, marital status, level of education, employment level, employment group, BMI, number of medical conditions, medications, number of dependents, homeschool responsibilities, smoking status, alcohol consumption, sleep quantity and sedentary behaviour) regressions were used to investigate relationships between PA, mental and physical work ability, absenteeism, and presenteeism. Logistic regression was used for binary outcomes, and ordinal logistic regression for ordered categorical outcomes. The proportional odds assumption was checked and was met for the ordered logistic regressions. Quantile linear regression for the median was used for continuous outcomes. Quantile regression was chosen, as the assumptions behind ordinary linear regression were not met, namely normality and constancy of variance of the residuals. Non-linear fits were also checked for the continuous covariates in the quantile regressions, but the Akaike Information Criterion (AIC) indicated that linear functional forms were better fits. To arrive at a parsimonious model of best fit, a backward elimination process based on the AIC was used. Mean and 95% confidence intervals (CIs) are reported unless otherwise indicated.

The Chi-Square test of association was used to investigate whether participants who reported meeting the MVPA guideline were more or less likely to be meeting the strength exercise guideline (and vice-versa), and to further investigate whether those with medical conditions or those in part-time employment were more or less likely to be meeting the MVPA or strength exercise guidelines, with the phi coefficient (correlation coefficient) indicating the effect size (0.1 = small, 0.3 = moderate, 0.5 = large). The number of participant responses for each outcome is reported in each table. Data processing, chi-square, and *t*-tests were conducted with Stata (version 16.1, StataCorp LLC, College Station, TX, USA). The regressions were carried out in freeware R [32]. The ordered logistic regressions were conducted with the polr function from the MASS library [33]. The quantile regressions were conducted with the quantreg library (R package version 5.67) [34].

## 3. Results

### 3.1. Participant Characteristics and Health-Related Behaviours

Participant characteristics are provided in Table 1. After checking against the inclusion criteria, 23 respondents were excluded from analyses as they were unemployed, leaving a final sample size of 433. Most participants were female (75%), aged 18–29 (24.7%) or 30–39 (30.3%) years, located in Victoria, Australia (51.4%), were Oceanian (51.3%), married (46.8%), had completed an undergraduate degree or higher (75.2%), had no dependents (63.5%), had not homeschooled children during the pandemic (72.5%), were employed full-time (69.7%), and worked as professionals (58%) (Table 1 and Table 2). Approximately half of the participants were of a normal weight (54%), and most were not taking any prescribed medications (67.8%) (Table 3). The most prevalent medical conditions reported were anxiety (14.1%), depression (11.1%), respiratory disease (10.0%), and hypertension (5.3%). Most participants were non-smokers (96.3%), and 76.9% reported consuming alcohol monthly, with 57.7% consuming alcohol weekly (Table 3). Participants slept a median 7 h per night and spent a median 7.9 h in sedentary behaviour each weekday. Over the week preceding the survey, most participants (60.5%) reported meeting the minimum MVPA guideline of ≥500 MET·min·week^−1^ [27]; however, less than half (45.9%) reported meeting the minimum strength exercise guideline of ≥2 days each week (Table 4). A little over one-third of the participants met both exercise guidelines (37.2%), while 30.8% met neither guideline.

### 3.2. Relationships between Exercise Participation and Participant Characteristics

Participants who met the MVPA guideline were significantly more likely to meet the strength exercise guideline, and vice-versa (*p* < 0.001; phi coefficient effect size = 0.39 (moderate)). Specifically, of the 244 participants who met the MVPA guideline, 150 (61.5%) also met the strength exercise guideline. Of the 185 participants who met the strength exercise guideline, 150 (81.1%) also met the MVPA guideline (Table 4). Compared to those without a medical condition, those with one or more medical conditions were less likely to meet the MVPA guideline (65.4% vs. 51.4%; *p* = 0.008; effect size = 0.14 (small)) but no less likely to meet the strength exercise guideline. There was no difference in the proportion of full-time and part-time employees meeting the strength exercise guideline (44.4% vs. 49.2%); however, the difference between the two groups meeting the MVPA guideline approached statistical significance (63.6% vs. 52.8%; *p* = 0.053; effect size = 0.10 (small)). Compared to full-time employees, more part-time employees were aged under 30 years (29.0% vs. 22.8%; *p*=0.028; effect size = 0.21 (small)), were female (84.0% vs. 71.2%; *p* = 0.018; effect size = 0.17 (small)), had one or more dependents for whom they provided primary care (50.0% vs. 30.7%; *p* = 0.002; effect size = 0.20 (small)), and were responsible for homeschooling one or more children during COVID-19 (36.9% vs. 23.4%; *p* = 0.006; effect size = 0.14 (small)), with no differences in marital status, medical conditions, or prescribed medications.

### 3.3. Associations between Exercise Participation and Work Ability

Current work ability (*n* = 349) was (median (IQR)) 20.0% (10–30%) below lifetime best, as were physical (20% (0–20%)) and mental work abilities (20% (20–40%)). Multivariate analyses determined that participants who met the MVPA guideline had (aOR (95% CI)) 1.80 (1.18–2.83; *p* = 0.007) times greater odds of reporting rather good or very good mental work ability, and 2.10 (1.34–3.29; *p* = 0.001) times greater odds of reporting rather good or very good physical work ability (Table 5). Significant univariate associations were observed between meeting the strength exercise guideline and odds of reporting higher physical (1.79 (1.20–2.68); *p* = 0.004) and mental (1.50 (1.03–2.19); *p* = 0.035) work ability. However, these associations were attenuated in the multivariate models for physical (1.46 (0.94–2.27); *p* = 0.092) and mental (1.43 (0.94–2.18); *p* = 0.088) work ability. There was no significant association between number of medical conditions and mental work ability; however, participants with three or more medical conditions (*n* = 20) were 80% less likely (aOR 0.20 (0.07–0.56); *p* = 0.002) to report rather good or very good physical work ability than participants with no medical conditions. Those in part-time employment were 56% less likely (aOR 0.44 (0.26–0.75); *p* = 0.002) to report rather good or very good mental work ability than those in full-time employment, but there was no significant association between employment fraction and physical work ability. Each 10% increase in daily sedentary behaviour was associated with a 31% reduction in the odds of reporting good or very good mental work ability (aOR 0.69 (0.59–0.81); *p* < 0.0001). Furthermore, a 10% increase in daily sedentary behaviour was associated with 3.68% higher median presenteeism (95% CI: 1.24–6.12%; *p* = 0.003). However, there was no significant association between sedentary behaviour and physical work ability (aOR = 0.72 (0.1–4.98); *p* = 0.74) or absenteeism (median (95% CI)) −1.67% ((−0.034, 0.0003); *p* = 0.053).

### 3.4. Associations between Exercise Participation, Absenteeism, and Presenteeism

Overall, participants (*n* = 349) reported working a median 4.0% (IQR: 0–21.0%) more hours over the previous month than what was expected of them (relative absenteeism) and reported a median 30.0% (IQR: 20.0–40.0%) lack of job performance (absolute presenteeism) over the same period. Compared to participants who did not meet the MVPA guideline, those who met the guideline reported a median 7.42% higher job performance (95% CI: 0.66–14.19%; *p* = 0.032) (Table 5). However, those who met the strength exercise guideline did not report different job performance to those who did not meet the guideline (median (95% CI)) +2.80% (−3.50–9.10%; *p* = 0.382). There were no differences in absenteeism for those meeting the MVPA (median (95% CI)) −0.33% (−5.00–4.00%; *p* = 0.880) or strength exercise guideline (median (95% CI)) 1.25% (−3.00–6.00%; *p* = 0.572) compared to those who did not meet the guidelines.

## 4. Discussion

Australian and New Zealand adult employees who reported meeting the MVPA guideline reported higher physical and mental work ability (i.e., occupational competence, health, and occupational virtues) and higher job performance compared to those who were not meeting the guideline during the early phase of the COVID-19 pandemic. Participants with one or more medical conditions were less likely to meet the MVPA guideline, while the difference between part-time (less likely) and full-time employees (more likely) approached statistical significance.

The association between meeting the MVPA guideline and higher work ability was expected, as previous research has reported that higher PA is associated with better cognitive and physical function and greater mental and physical wellbeing in healthy adults in the workplace [13,14,35]. Our finding that participants who met the MVPA guideline reported higher job performance and work ability during the COVID-19 pandemic compared to those who did not meet the guideline is important, as other research has reported reduced job performance (i.e., greater presenteeism) in adults who transitioned from the workplace to working either fully or partly from home during this time [36]. Critically, though, the potential effect for PA to influence job performance during work from home was not investigated [36], as was the case in our study. Furthermore, software professionals working from home during the pandemic reported that distractions, boredom, and the need for competence were negatively associated with self-reported job performance, but in contrast to our findings, there was no association between job performance and PA [37]. Our findings suggest that engagement in PA can support employee job performance in changing work environments, which is an important consideration for employers.

General work ability was 20% below lifetime best, and participants reported working 4.0% hours more over the past month than usually required (i.e., overtime) but reported a 30.0% lack of job performance, which may reflect job-related [5] or other stress and may lead to poorer health outcomes over time [38]. Factors negatively affecting work ability have been identified as a lack of leisure time, vigorous PA, poor musculoskeletal health, older age, high mental work demands, and poor work environment [39]. During COVID-19, many employees were required to work from home, resulting in increased distractions and fewer peer-to-peer social interactions, potentially negatively affecting wellbeing during this time [5,37]. The pandemic also appears to have negatively impacted mental health, which could be a factor affecting work performance [1,4,5]. In addition, COVID-19 may have compounded already burdened employees with additional personal responsibilities while working from home, such as homeschooling or caring for relatives. For example, a survey of US employees working from home during the COVID-19 pandemic found that a reduction in physical and mental wellbeing was associated with several factors, including less exercise, having at least one child at home, distractions while working, less co-worker communication, higher workload, having to adjust work hours, and lower satisfaction with indoor workspace set-up [35]. Participants in our study may have been experiencing similar burdens, as they reported a 30% lack of job performance despite working more hours than was expected of them, noting that those who with higher MVPA participation reported lower presenteeism.

Part-time employees were less likely to meet the MVPA guideline than full-time employees and were less likely to report a rather good or very good mental work ability. Part-time employees were more likely to be female and reported having additional responsibilities; specifically, they were more likely to have caring and homeschooling responsibilities, which could have reduced their opportunities for PA participation. Given the evidence that increasing PA improves physical and mental work ability [20,21], part-time employees might require additional support to promote engagement in PA and exercise, particularly if they are not commuting to the workplace. Furthermore, those who had one or more medical conditions were less likely to meet the MVPA guidelines, while people with three or more medical conditions were less likely to report a rather good or very good physical work ability. Reports of severity of COVID-19 infection in individuals with existing medical conditions such as diabetes and obesity may have deterred individuals from completing MVPA for want of lowering their exposure risk, or those with medical conditions may have been less physically active as a result of their condition(s). Either way, employees with medical conditions might require additional exercise behaviour change support or education on how to exercise safely, particularly in these circumstances. 

The amount of weekday sedentary behaviour that people reported (~7.9 h) was similar to that of the average healthy adult population (median 8.2 h) prior to the COVID-19 pandemic [40]. Previous research in a similar population demonstrated that employees spend around 77% (~6.6 h) of their working hours and almost two-thirds (~4.3 h) of non-working hours each weekday in sedentary behaviour [41], and sedentary time was potentially 50% greater in those working from home compared to those working at the workplace during the COVID-19 pandemic [3]. In our study, the likelihood of reporting poorer mental work ability and reduced job performance increased with increases in sedentary time. 

The proportion of participants in this study who reported meeting the MVPA and strength exercise guidelines was higher than that reported in adult Australian and international populations prior to COVID-19 [9,10,11]. Other data have indicated that some people may have engaged in more PA because they had more time due to not having to commute to work and recognised the need to participate in regular PA during the pandemic [2]. Of the 185 participants who met the strength exercise guideline, 81.1% also met the MVPA guideline. This finding suggests that promoting strength exercise participation might be an effective strategy to increase adherence to both the MVPA and strength exercise guidelines when movement and social restrictions are in place. With appropriate education, strength exercise in the form of using body weight as resistance can be easily completed in the home, allowing people with existing medical conditions to restrict potential exposure to COVID-19 while increasing their PA participation.

It must be acknowledged that the results are only generalisable to the sample included in the survey and not the wider working population. Furthermore, there are limitations with the use of the self-reporting tools to collect data on PA, and the IPAQ measures PA over the past 7 days, whereas presenteeism and absenteeism were measured over the past month. The specific restrictive measures implemented across Australia and New Zealand differed between jurisdictions throughout the survey period, and it is possible that movement and social restrictions changed between the earliest date requested to consider activities in the survey tools and when the respondent completed the survey. Therefore, it is not clear how the current level of restriction in relation to any previous restrictions influenced outcomes, and a decision was made not to include degree of restriction as a covariate. 

## 5. Conclusions

During the early phase of the COVID-19 pandemic, with various movement and social restrictions placed on adult employees in Australia and New Zealand, meeting the MVPA guideline was associated with greater odds of reporting good physical and mental work ability and higher job performance. Furthermore, higher sedentary behaviour was associated with lower reported mental work ability and job performance. These findings support the idea that employees should be provided opportunities and encouraged to participate in regular exercise, as this has the potential to benefit both employers (higher productivity) and employees (higher work ability and health outcomes). Physical work ability and MVPA were lower in those with existing medical conditions, while mental work ability and MVPA were lower in part-time employees. These populations may require additional physical activity health behaviour support to begin and sustain regular exercise participation, and future interventions should account for this in their design. To ensure acceptable levels of presenteeism and promote good work ability, employers should promote and educate employees on safe exercise strategies at the workplace and when working from home to assist with managing work and general stressors during a changing and uncertain global environment.

## Figures and Tables

**Table 1:** Descriptive characteristics of the participant sample.

Variable	*n*	(%)
Total participant sample	433	100
Gender		
Male	101	23.3
Female	325	75.1
Non-binary	3	0.7
Other	4	0.9
Age (years)		
18–29	107	24.7
30–39	131	30.3
40–49	95	21.9
50–59	75	17.3
60+	25	5.8
Residence		
New Zealand	17	3.7
Australia	416	96.3
Victoria	214	51.4
Western Australia	160	38.5
New South Wales	33	7.9
Queensland	3	0.7
South Australia	3	0.7
Tasmania	3	0.7
Ethnicity		
Oceanian	222	51.3
European	166	38.3
Asian	25	5.8
Other	20	4.6
Marital Status		
Married	198	46.8
Never married	103	24.3
De facto	90	21.3
Widowed/Divorced/Separated	32	7.6
Level of Education		
Postgraduate degree/Diploma	185	42.8
University/College degree	140	32.4
Certificate I-IV/Diploma/Advanced Diploma	66	15.2
Completed secondary school	33	7.6
Did not complete secondary school/other	8	1.9
Number of Dependents		
0	273	63.5
1 or 2	126	29.3
3+	31	7.2
Homeschooled children during COVID-19		
Yes	118	27.5
No	311	72.5

BMI, Body Mass Index (underweight, <18.5 kg·m^−2^; normal range, 18.5–24.99 kg·m^−2^; overweight, 25.00–29.99 kg·m^−2^; obese, ≥30.00 kg·m^−2^); CI, Confidence Interval; IQR, Interquartile range.

**Table 2:** Employment characteristics of the participant sample.

Variable	*n*	(%)
Employment Fraction		
Full-time	302	69.7
Part-time	131	30.3
Employment Group		
Professionals	251	58.0
Clerical and Administration Workers	61	14.1
Managers	57	13.2
Community and Personal Services Workers	23	5.3
Sales Workers	16	3.7
Technicians and Trades Workers	13	3.0
Other	12	2.8

**Table 3:** Health status and health-related behaviours of the participant sample.

Variable	*n*	(%)
BMI Classification		
Underweight	4	1.0
Normal weight	223	54.0
Overweight	131	31.7
Obese	55	13.3
Medical Conditions		
0	276	63.9
1 or 2	136	31.4
3+	20	4.6
Prescribed Medications		
Yes	139	32.2
No	293	67.8
Smoking Status		
Not a smoker	417	96.3
Current smoker	16	3.7
Alcohol Consumption		
4–7 times per week	47	10.9
2–3 times per week	110	25.5
Once per week	92	21.3
2–3 times per month	54	12.5
Once per month	29	6.7
A few times per year	52	12.0
None in the last year	17	3.9
Never	31	7.2
Sleep Quantity: Median (IQR)	7.0 (6.5–8.0)	
Sedentary Behaviour: Median (IQR)	7.9 (6.0–10.1)	

Current smoker (current smoker or quit in the last six months); Medical conditions (number of diagnosed medical conditions); Prescribed medications (currently taking any prescribed medications); Sedentary behaviour (hours per weekday spent sitting); Sleep quantity (average hours per night).

**Table 4:** Aerobic and strength exercise participation.

	Strength Exercise Participation: *n* (%)		
Moderate-vigorous aerobic exercise participation, *n* (%)	<2 days·wk^−1^	≥2 days·wk^−1^	Total
<500 MET-min·wk^−1^	124 (30.8)	35 (8.7)	159 (39.5)
≥500 MET-min·wk^−1^	94 (23.3)	150 (37.2)	244 (60.5)
Total	218 (54.1)	185 (45.9)	403 (100.0)

MET, Metabolic equivalent of energy expenditure.

**Table 5:** Multivariate regression results.

	Estimated Difference	95% CI	*p* Value
Meeting vs. not meeting the MVPA guideline			
Physical Work Ability	2.10	1.34–3.29	0.001
Mental Work Ability	1.80	1.18–2.83	0.007
Relative Absenteeism	−0.33%	−5.00–4.00%	0.880
Absolute Presenteeism	7.42%	0.66–14.19%	0.032
Meeting vs. not meeting the strength exercise guideline			
Physical Work Ability	1.46	0.94–2.27	0.092
Mental Work Ability	1.43	0.94–2.18	0.088
Relative Absenteeism	1.25%	−3.00–6.00%	0.572
Absolute Presenteeism	2.80%	−3.50–9.10%	0.382

CI, Confidence Interval; *n* = 349. Adjusted odds ratio reported for physical work ability and mental work ability. Median reported for absenteeism and presenteeism.

## Data Availability

The data presented in this study are openly available in La Trobe University’s Institutional Repository (OPAL) at doi:10.26181/619d8d4a12349.

## Appendix A

**Table A1:** STROBE Checklist.

	Item No.	Recommendation	Page No.
Title and abstract	1	(a) Indicate the study’s design with a commonly used term in the title or the abstract	1
		(b) Provide in the abstract an informative and balanced summary of what was done and what was found	1
Introduction			
Background/rationale	2	Explain the scientific background and rationale for the investigation being reported	2
Objectives	3	State specific objectives, including any prespecified hypotheses	2
Methods			
Study design	4	Present key elements of study design early in the paper	2–3
Setting	5	Describe the setting, locations, and relevant dates, including periods of recruitment, exposure, follow-up, and data collection	2–3
Participants	6	Cross-sectional study—Give the eligibility criteria, and the sources and methods of selection of participants	3
Variables	7	Clearly define all outcomes, exposures, predictors, potential confounders, and effect modifiers. Give diagnostic criteria, if applicable	3–4
Data sources/ measurement	8 *	For each variable of interest, give sources of data and details of methods of assessment (measurement). Describe comparability of assessment methods if there is more than one group	3–4
Bias	9	Describe any efforts to address potential sources of bias	2–4
Study size	10	Explain how the study size was arrived at	3
Quantitative variables	11	Explain how quantitative variables were handled in the analyses. If applicable, describe which groupings were chosen and why	4–5
Statistical methods	12	(a) Describe all statistical methods, including those used to control for confounding	4–5
		(b) Describe any methods used to examine subgroups and interactions	4–5
		(*c*) Explain how missing data were addressed	5
		Cross-sectional study—If applicable, describe analytical methods taking account of sampling strategy	4–5
		(*e*) Describe any sensitivity analyses	4–5
Results			
Participants	13 *	(a) Report numbers of individuals at each stage of study—e.g., numbers potentially eligible, examined for eligibility, confirmed eligible, included in the study, completing follow-up, and analysed	5
		(b) Give reasons for non-participation at each stage	5
		(c) Consider use of a flow diagram	N/A
Descriptive data	14 *	(a) Give characteristics of study participants (e.g., demographic, clinical, social) and information on exposures and potential confounders	5–7
		(b) Indicate number of participants with missing data for each variable of interest	5–8
Outcome data	15 *	Cross-sectional study—Report numbers of outcome events or summary measures	5–8
Main results	16	(a) Give unadjusted estimates and, if applicable, confounder-adjusted estimates and their precision (e.g., 95% confidence interval). Make clear which confounders were adjusted for and why they were included	8
		(b) Report category boundaries when continuous variables were categorized	N/A
		(c) If relevant, consider translating estimates of relative risk into absolute risk for a meaningful time period	N/A
Other analyses	17	Report other analyses done—e.g., analyses of subgroups and interactions, and sensitivity analyses	7–8
Discussion			
Key results	18	Summarise key results with reference to study objectives	9
Limitations	19	Discuss limitations of the study, taking into account sources of potential bias or imprecision. Discuss both direction and magnitude of any potential bias	10
Interpretation	20	Give a cautious overall interpretation of results considering objectives, limitations, multiplicity of analyses, results from similar studies, and other relevant evidence	10–11
Generalisability	21	Discuss the generalisability (external validity) of the study results	10
Other information			
Funding	22	Give the source of funding and the role of the funders for the present study and, if applicable, for the original study on which the present article is based	11

Reprinted from von Elm et al. (2007) [24]. * Give information separately for cases and controls in case-control studies and, if applicable, for exposed and unexposed groups in cohort and cross-sectional studies. **Note:** An Explanation and Elaboration article discusses each checklist item and gives methodological background and published examples of transparent reporting. The STROBE checklist is best used in conjunction with this article (freely available on the Web sites of PLoS Medicine at, Annals of Internal Medicine at http://www.annals.org/ (accessed on 21 August 2021), and Epidemiology at http://www.epidem.com/ (accessed on 21 August 2021)). Information on the STROBE Initiative is available at www.strobe-statement.org (accessed on 21 August 2021).
